# MicroRNA‐501‐3p inhibits the proliferation of kidney cancer cells by targeting WTAP

**DOI:** 10.1002/cam4.4157

**Published:** 2021-09-30

**Authors:** Liujia He, Shiming Chen, Yufan Ying, Haiyun Xie, Jiangfeng Li, Xueyou Ma, Weiyu Wang, Haixiang Shen, Xiao Wang, Xiangyi Zheng, Liping Xie

**Affiliations:** ^1^ Department of Urology School of Medicine The First Affiliated Hospital Zhejiang University Hangzhou China

**Keywords:** microRNA‐501‐3p, proliferation, renal cell carcinoma, WTAP

## Abstract

**Background:**

Emerging evidence suggests that miR‐501‐3p plays an important role in the pathogenesis and progression of various carcinomas. However, its role and underlying mechanisms in renal cell carcinoma (RCC) remain to be elucidated.

**Methods:**

Quantitative RT‐PCR, western blot, and bioinformatics methods were used to evaluate the expression of miR‐501‐3p and Wilms’ tumor 1‐associating protein (*WTAP*) in RCC cell lines and clinical tissues. The effects of miR‐501‐3p on the proliferation of RCC cells were investigated using flow cytometric, colony formation, and CCK8 assays. The target gene of miR‐501‐3p was confirmed by western blotting, qRT‐PCR, and dual‐luciferase reporter assays. The levels of RNA methylation with N6‐methyladenosine (m^6^A) following miR‐501‐3p overexpression or knockdown of its target gene were quantified using a dot‐blot assay.

**Results:**

miR‐501‐3p expression was significantly downregulated in human RCC cell lines and tissues. In contrast, its overexpression markedly inhibited cancer cell proliferation in vitro by inducing G1 phase arrest. Moreover, *WTAP* was verified as a direct target gene of miR‐501‐3p. *WTAP* gene knockdown alone efficiently produced the same cancer‐inhibiting effects as miR‐501‐3p overexpression, with the level of m^6^A in RCC cells being decreased under both scenarios. The intermolecular interaction between miR‐501‐3p and *WTAP* was further substantiated by rescue experiments.

**Conclusion:**

RCC progression is regulated via the miR‐501‐3p/WTAP/CDK2 axis and is inhibited by the overexpression of miR‐501‐3p.

## Graphical abstract

miR‐501‐3p was significantly down‐regulated in human RCC cell lines and tissues. Moreover, WTAP was confirmed as the direct target gene of miR‐501‐3p. RCC progression is regulated via the miR‐501‐3p/WTAP/CDK2 axis and is inhibited by the overexpression of the microRNA.

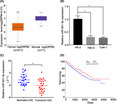

## 1 INTRODUCTION

Renal cell carcinoma (RCC), a common malignancy of the urinary system, has gradually increased in incidence owing to smoking habits and dietary changes in people worldwide.^1^ Globally, RCC accounted for approximately 403,000 new cases and 175,000 deaths in 2018.^2^ In 2020, approximately 73,800 patients developed RCC in the United States, and 14,800 deaths were related to the disease.^3^ Currently, surgery is the preferred treatment for localized tumors. However, because approximately 30%–40% of patients present with metastasized RCC by the time they are first diagnosed,^4^, ^5^ there is a crucial need to develop more effective early diagnostic and therapeutic approaches for RCC.

MicroRNAs (miRNAs) regulate the expression of target genes at the post‐transcriptional level. Numerous studies have shown that miRNAs are involved in the regulation of many cellular processes, such as cell proliferation, development, and apoptosis.^6^, ^7^, ^8^ In RCC, miRNAs inhibit the pathogenesis of the disease by targeting more than one key gene and are dysregulated during disease progression.^9^ In particular, miR‐182‐5p, miR‐452‐5p, miR‐381‐3, and miR‐30a‐5p are involved in the regulation of the cell cycle, apoptosis, and metastasis of RCC cells.^10^, ^11^, ^12^, ^13^

Recently, miR‐501‐3p has been suggested to be involved in many diseases, and research on it has gradually increased. Hara *et al*. found that miR‐501‐3p was downregulated in the serum of patients with Alzheimer’s disease, and its low expression was significantly related to the mild mental status score of individuals examined.^14^ In another study, miR‐501‐3p directly targeted the RAS oncogene *RAP1A* to repress cell proliferation in non‐small cell lung cancer.^15^ MiR‐501‐3p also directly targets the Lin‐7 homolog A (*LIN7A*) gene to repress the development of hepatocellular carcinoma,^16^ and its direct regulation of the Fos proto‐oncogene (*FOS*), myogenic differentiation (*MyoD*) gene, and MyoD family inhibitor (*MDFI*) gene may be involved in the pathogenesis of skeletal muscle metabolic diseases.^17^ However, the expression pattern and biological function of miR‐501‐3p in RCC are yet to be elucidated. Therefore, this study aimed to investigate whether miR‐501‐3p is involved in the regulation of RCC pathogenesis and to determine the molecular mechanisms underlying this process.

## 2 MATERIALS AND METHODS

### 2.1 Cell lines and cell culture

HK‐2, 786‐O, and Caki‐1 cell lines were obtained from the Cell Bank of the Chinese Academy of Sciences. Cell culture experiments were conducted according to the institutional guidelines.

### 2.2 Human clinical samples

From January 2013 to October 2013, human RCC tissues were collected from patients undergoing radical nephrectomy and matched to adjacent non‐neoplastic renal tissues. In total, 24 samples were obtained from the patients after obtaining their informed consent. The study was approved by the ethics committee of the First Affiliated Hospital, Zhejiang University School of Medicine. The samples were stored in liquid nitrogen until use. Clinical information of patients is shown in Table S1.

### 2.3 Cancer genome atlas database

The Cancer Genome Atlas (TCGA; https://cancergenome.nih.gov/) database was used to evaluate the expression pattern of miR‐501‐3p in RCC. The Database for Annotation, Visualization, and Integrated Discovery (DAVID; http://david.abcc.ncifcrf.gov) was used to query the Gene Ontology terms for the predicted miRNA targets. The associated integrated databases UALCAN (http://ualcan.path.uab.edu/) and starBase (http://starbase.sysu.edu.cn/) were used to analyze the expression patterns of Wilms tumor 1‐associating protein (WTAP) and miR‐501‐3p in RCC. The downstream target genes were predicted using the miRWalk (http://miwalk.umm.uni‐heidelberg.de/), starBase, and miRanda (http://www.miRanda.org) databases. Overall survival relative to miR‐501‐3p and WTAP expression in RCC was analyzed using OncoLnc (http://www.oncolnc.cn).

### 2.4 Reagents and transfection

The *WTAP* overexpression plasmid (pWTAP) and control plasmid pNull were purchased from GeneChem Company. Small interfering RNAs (siRNAs) were purchased from GeneChem. The corresponding sequences are listed in Table S2. To forestall off‐target effects, three dissimilar siRNAs were merged for the co‐transfection of cancerous cells in all trials. Transfection was performed using Lipofectamine 2000 reagents (Invitrogen) according to the manufacturer’s protocol.

### 2.5 RNA isolation and quantitative real‐time PCR

RNAiso Plus (Takara) was used to extract RNA from RCC cells and clinical tissues according to the manufacturer’s instructions. The PrimeScript miRNA cDNA Synthesis Kit (Takara) and PrimeScript RT Reagent Kit (Takara) were used to reverse transcribe the isolated RNA. The resultant cDNA was amplified with the ABI 7500 Fast Real‐time PCR System (Applied Biosystems), and the relative expression levels of mRNA and miRNA were determined using SYBR Green (Takara). These levels were then compared using the 2^△△Ct^ method after normalization to the expression of the reference genes glyceraldehyde 3‐phosphate dehydrogenase (*GAPDH*) and *U6 small nuclear RNA*.

### 2.6 Colony formation assay

Five hundred transfected 786‐O and Caki‐1 cells were seeded in six‐well plates per well and cultured for 2 weeks. Colony counts and rates were analyzed after the cells were treated with methanol and 0.2% crystal violet.

### 2.7 Cell viability assay

The 786‐O and Caki‐1 cells were seeded in 96‐well plates, and when the confluence reached 30%–50% per well, the merged siRNA was used to transfect the cells at different concentrations. After 48 or 72 h of treatment, counting kit‐8 (Dojindo) was used to detect the cell viability according to the manufacturer’s protocol.

### 2.8 Cell cycle analysis

The 786‐O and Caki‐1 cells were digested and washed with phosphate buffer saline and then fixed overnight with 75% ethanol at 4℃. A Cell cycle staining Kit (Multi Sciences) was used to stain the DNA. After 60 min, cell cycle assays were performed using flow cytometry (BD LSRII Flow Cytometer System, BD Biosciences). ModFit LT 3.2 software (Verity Software House) was used to analyze the data.

### 2.9 Western blot analysis

The proteins were extracted in radioimmunoprecipitation assay (RIPA) lysis buffer, and their relative concentrations were quantified using the BCA Protein Assay kit (Beyotime). Proteins were loaded onto 10% SDS‐polyacrylamide gels and subjected to electrophoresis. The wet transfer method was used to transfer the completely separated proteins to PVDF membranes. Fat‐free milk was used to block the proteins for 1 h and then the membranes were incubated with the primary antibody (1:1000 dilution) at 4℃ for 14–16 h. After washing with TBS‐Tween buffer, the membranes were incubated with the secondary antibody (1:5000 dilution) at 20–25℃ for 1 h. After the second wash with TBS‐Tween buffer, protein expression levels were detected using a chemiluminescence system (Pierce Biotechnology Inc.). Antibodies used in this study were as follows: anti‐CDK2, anti‐CDK4, anti‐CDK6, anti‐CCND1, anti‐CCNE1, anti‐WTAP, and anti‐GAPDH.

### 2.10 Dual‐luciferase reporter assay

Oligonucleotides containing either the wild‐type or mutated 3′ untranslated region (3′‐UTR) of the potential target genes of miR‐501‐3p were designed by us and produced by Sangon.

After the annealing step, the specific oligonucleotide was inserted into a double‐stranded segment between the SacI and SalI sites of the pmirGLO Dual‐Luciferase miRNA Target Expression Vector (Promega). The insertion was confirmed by DNA sequencing. RCC 786‐O cells that had been inoculated into 24‐well plates were then co‐transfected with 50 nM miR‐501‐3p mimic (or NC) and 100 ng of the pmirGLO reporter. The dual‐luciferase reporter assay was then used to quantify the relative luciferase activity in the transfected cells after 48 h.

### 2.11 Dot‐blot assay of RNA methylation with N6‐methyladenosine

RNA was isolated from various cell lines using RNAiso plus (Takara), and its concentration was adjusted to 50 ng/μl with 36 μl of RNase‐free water. The RNA secondary structure was determined using RNA incubation buffer (mixture of MOPS, formamide, and formaldehyde) and treatment with ice‐cold 20 × saline‐sodium citrate solution (Sigma‐Aldrich), RNA samples (200 ng) were loaded onto an N+ membrane (GE Healthcare) in a dot blot apparatus (Bio‐Rad Laboratories). To guarantee that an equal amount of total RNA was scanned, the RNA was bound to the membrane by UV cross‐linking before being stained with 0.02% methylene blue (Sigma‐Aldrich). Thereafter, the membrane was blocked with skimmed milk, incubated with the m^6^A antibody (1:2000, Synaptic Systems), incubated with the secondary antibody, and finally exposed to the ChemiDoc MP imaging system (Bio‐Rad).

### 2.12 Statistical analysis

All data are expressed as the mean ± standard deviation (SD). The two‐tailed Student’s *t*‐test was used to assess the statistical differences between the two estimates. All analyses were performed using GraphPad Prism version 7. Significance was defined as a two‐tailed value of *p *< 0.05.

## 3 RESULTS

### 3.1 miR‐501‐3p is downregulated in RCC

**FIGURE 1:**
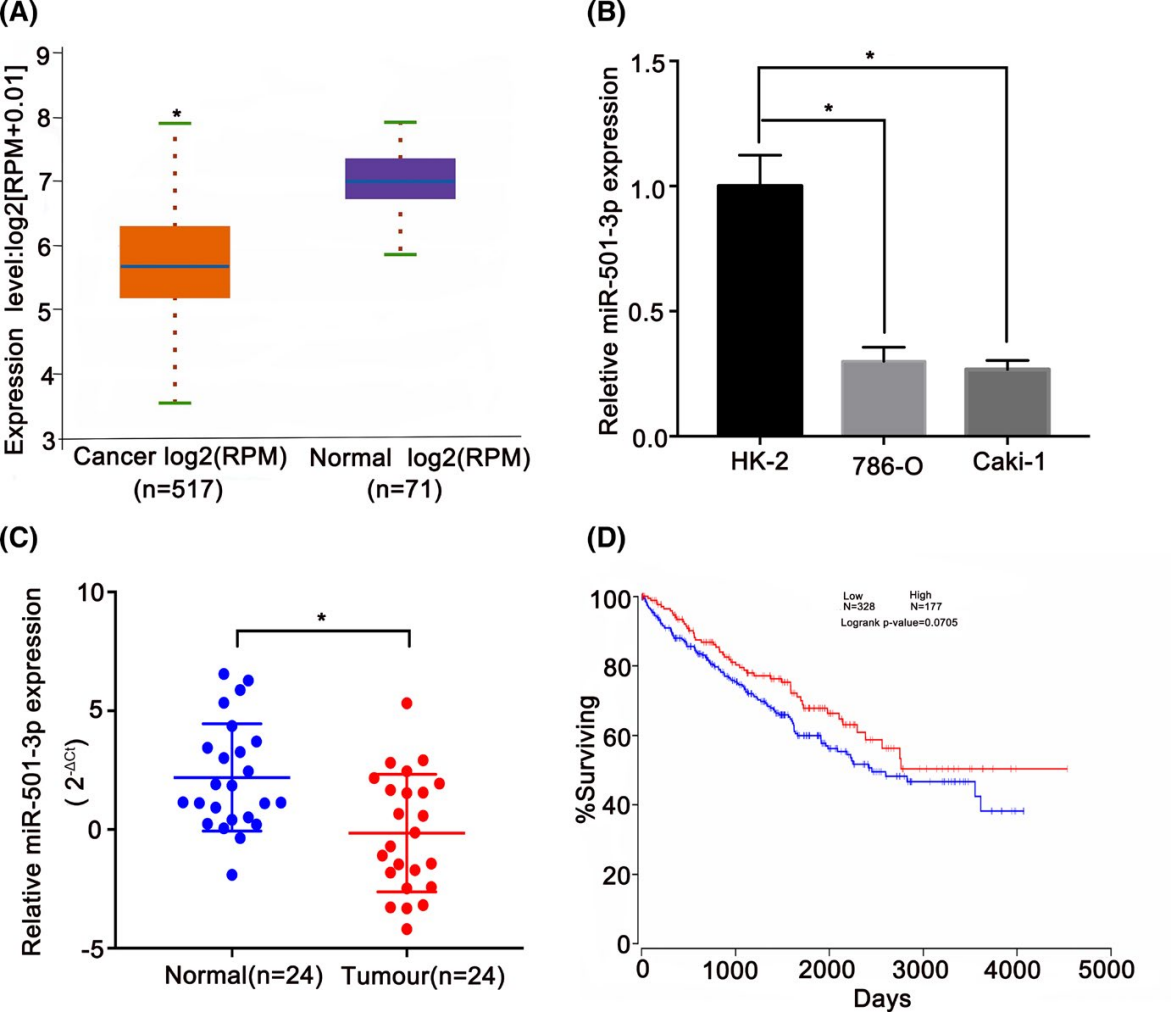
miR‐501‐3p is downregulated in renal cell carcinoma (RCC). (A) StarBase database revealed that the expression of miR‐501‐3p in RCC was markedly decreased in RCC tissues than in the normal renal tissues (fold change = 0.46, *p* < 0.0001). (B) Compared with HK‐2, miR‐501‐3p was downregulated in RCC cell lines. (C) miR‐501‐3p expression in clinical samples of RCC was confirmed by quantitative real‐time PCR (qRT‐PCR). (D) Kaplan–Meier plot survival analysis showed that high expression of miR‐501‐3p was associated with higher overall survival of RCC (*p *= 0.0705). *p < 0.05

According to the TCGA database, the expression of miR‐501‐3p was markedly downregulated in RCC tissues compared to that in normal renal tissues (Figure 1A). Additionally, the qRT‐PCR analysis revealed that miR‐501‐3p expression levels were significantly lower in cancerous cells than in non‐cancerous cells (Figure 1B). To further confirm the expression pattern of miR‐501‐3p in RCC, 24 pairs of clinical RCC samples were also quantified by qRT‐PCR, and the expression level of miR‐501‐3p was found to be downregulated in the cancerous tissues only (Figure 1C). Moreover, patients with a higher level of miR‐501‐3p expression had a longer overall survival time than those with a lower expression level of this miRNA (*p *= 0.0705, Figure 1D). In summary, these findings suggest that miR‐501‐3p has a potential antitumor function in RCC.

### 3.2 miR‐501‐3p overexpression inhibits kidney cancer cell proliferation

**FIGURE 2:**
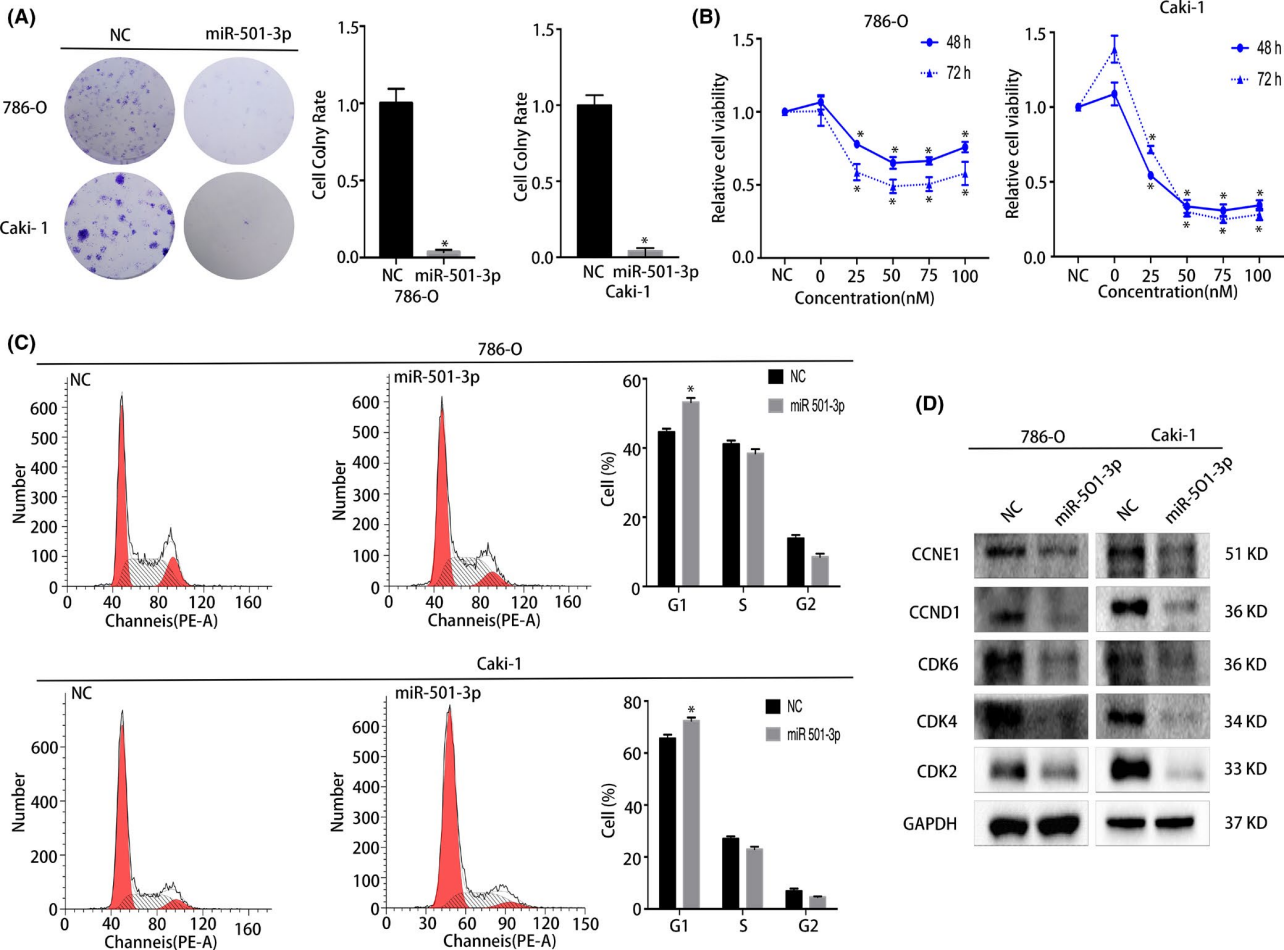
miR‐501‐3p overexpression repressed the proliferation of renal cell carcinoma (RCC) cell lines. (A) Colony formation assay, miR‐501‐3p mimic‐treated group had a lower rate of colony formation than the NC group (representative wells are presented). (B) CCK8 assay demonstrated the diminished cell viability upon transfection with the miRNA mimic in RCC cell lines. (C) Cell cycle analysis revealed that the percentage of cells arrested in the G1 phase in the miR‐501‐3p mimic‐treated group was significantly higher than that in the NC group (representative histograms are presented). (D) Western blots showed the changes in proteins associated with the cell cycle of miR‐501‐3p‐overexpressed RCC cell lines. Error bars represent the standard deviation (SD) obtained from three independent experiments. *p < 0.05

To determine the function of miR‐501‐3p, we designed a miR‐501‐3p mimic for transfection into the RCC cell lines to upregulate its levels in these cells. The proliferation ability of the transfected cells was determined using the colony formation assay, and the results showed that the upregulation of miR‐501‐3p notably diminished the growth of both RCC cell lines (Figure 2A). Additionally, the CCK8 assay further revealed the inhibition of cell viability at different times and siRNA transfection concentrations (Figure 2B). To further investigate the mechanisms of proliferation suppression, a flow cytometric assay was performed, which showed that the overexpression of miR‐501‐3p led to G1 phase arrest (Figure 2C). Coincidentally, the protein expression levels of the corresponding G1/S transition regulators (CDK2, CDK4, CDK6, CCND1, and CCNE1) were reduced, as revealed by western blot analysis (Figure 2D).

### 3.3 *WTAP* is the direct target gene of miR‐501‐3p

**FIGURE 3:**
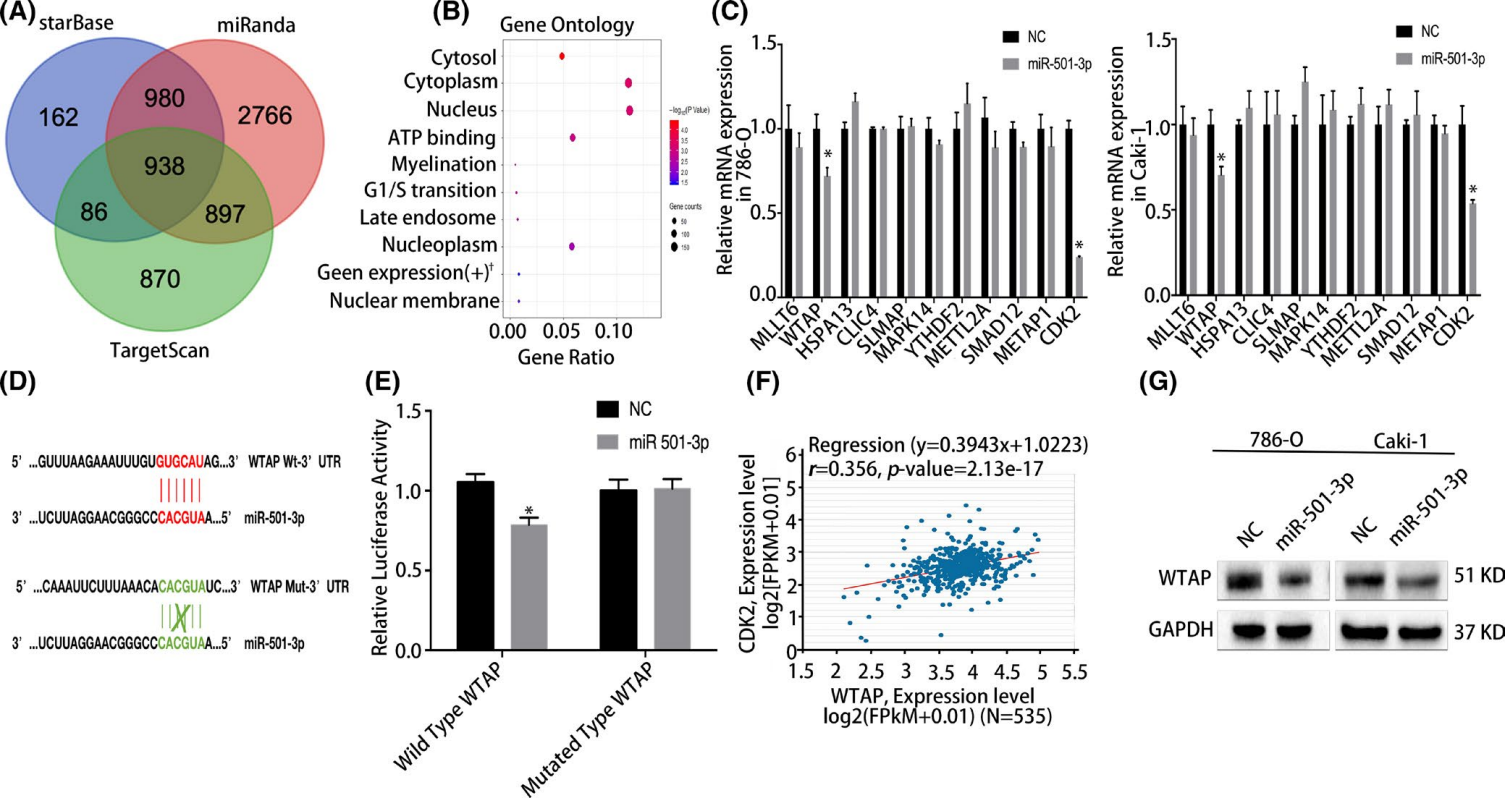
miR‐501‐3p targets the *WTAP* gene in renal cell carcinoma (RCC). (A) TargetScan, starBase, and miRanda databases were used to predict potential downstream targets of miR‐501‐3p. (B) Gene Ontology enrichment analysis (representative pathways are presented). (C) Quantitative real‐time PCR (qRT‐PCR) experiments showed that *WTAP* was significantly downregulated at the RNA level in RCC cell lines after transfection with the miR‐501‐3p mimic. (D) Schematic diagram of the miR‐501‐3p‐targeting region of *WTAP* with seed matching. (E), Dual‐luciferase reporter assay demonstrated that miR‐501‐3p notably inhibited the luciferase activity of vectors that carried the 3′‐UTR of WTAP in the 786‐O cell line. (F) Regression analysis of *WTAP* and *CDK2* in RCC, date from starBase database (*r *= 0.356, *p *= 2.13e–17). (G) Western blots confirmed that *WTAP* was notably downregulated after transfected with the miR‐501‐3p mimic. Error bars represent the standard deviation (SD) obtained from three independent experiments; †, positive regulation of gene expression. *p < 0.05

The direct target genes of miR‐501‐3p were predicted using the starBase, TargetScan, and miRanda databases. In total, 938 candidate genes were identified (Figure 3A) and subsequently subjected to Gene Ontology enrichment analysis. The results revealed that the candidate target genes were mainly expressed in the cytoplasm, nucleus, and cell cycle pathway (Figure 3B). Several candidate genes were selected for the qRT‐PCR evaluation of their mRNA levels in cells transfected with the miR‐501‐3p mimic. Consequently, two candidate target genes (*WTAP* and *CDK2*) were found to have decreased mRNA levels in RCC cell lines (Figure 3C). Consistently, western blot analysis confirmed the depletion of *WTAP* at the protein level in cells expressing the miR‐501‐3p mimic (Figure 3G). To further validate these results, a dual‐luciferase reporter assay was performed. The results showed that the luciferase activity in the cells co‐transfected with the wild‐type 3′‐UTR of *WTAP* was reduced, whereas there was no change in activity in the mutated 3′‐UT (Figure 3D,E). These findings suggest that miR‐501‐3p binds directly to the predicted binding site of *WTAP*.

### 3.4 *WTAP* is upregulated in RCC

**FIGURE 4:**
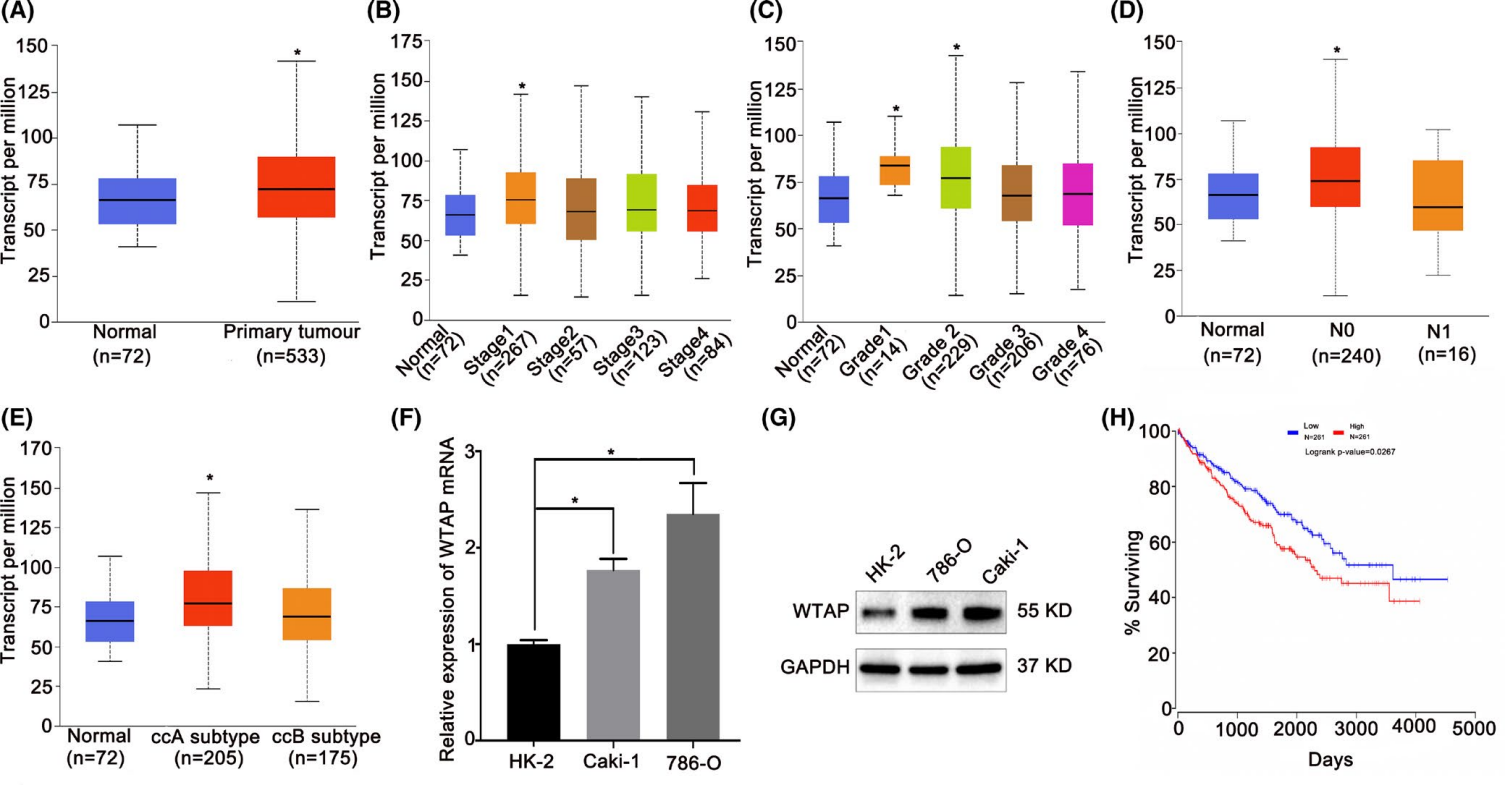
*WTAP* is highly expressed in renal cell carcinoma (RCC). (A) The UALCAN database indicated that *WTAP* expression was significantly higher in RCC than in normal kidney tissues (*p* = 0.0032204). (B–E) The TCGA database showed differences in mRNA expression of *WTAP* in RCC among stages, grades, nodal metastasis status, and subtypes. (F) At the mRNA level, the expression of *WTAP* was markedly increased in RCC cells. (G) Western blots indicated the upregulated expression of *WTAP* in RCC cell lines compared to HK‐2 cells. GAPDH was used for normalization. (H) Kaplan–Meier survival analysis indicated that patients with high *WTAP* expression had a lower survival time than those with low RCC expression (*p *= 0.0267). *p < 0.05

Analysis of the TCGA database revealed that *WTAP* was markedly upregulated in RCC tissues than in normal tissues (Figure 4A–E). The qRT‐PCR analysis further verified that the miR‐501‐3p expression level was significantly higher in the RCC cell lines than in the HK‐2 cell line (Figure 4F). These same findings were demonstrated by the western blot (Figure 4G). Moreover, the prognosis of patients with high *WTAP* expression was worse than that of patients with low expression (*p *= 0.00257, Figure 4D). These findings suggest that *WTAP* promotes the progression of RCC.

### 3.5 *WTAP* depletion impairs the proliferation of kidney cancer cells

**FIGURE 5:**
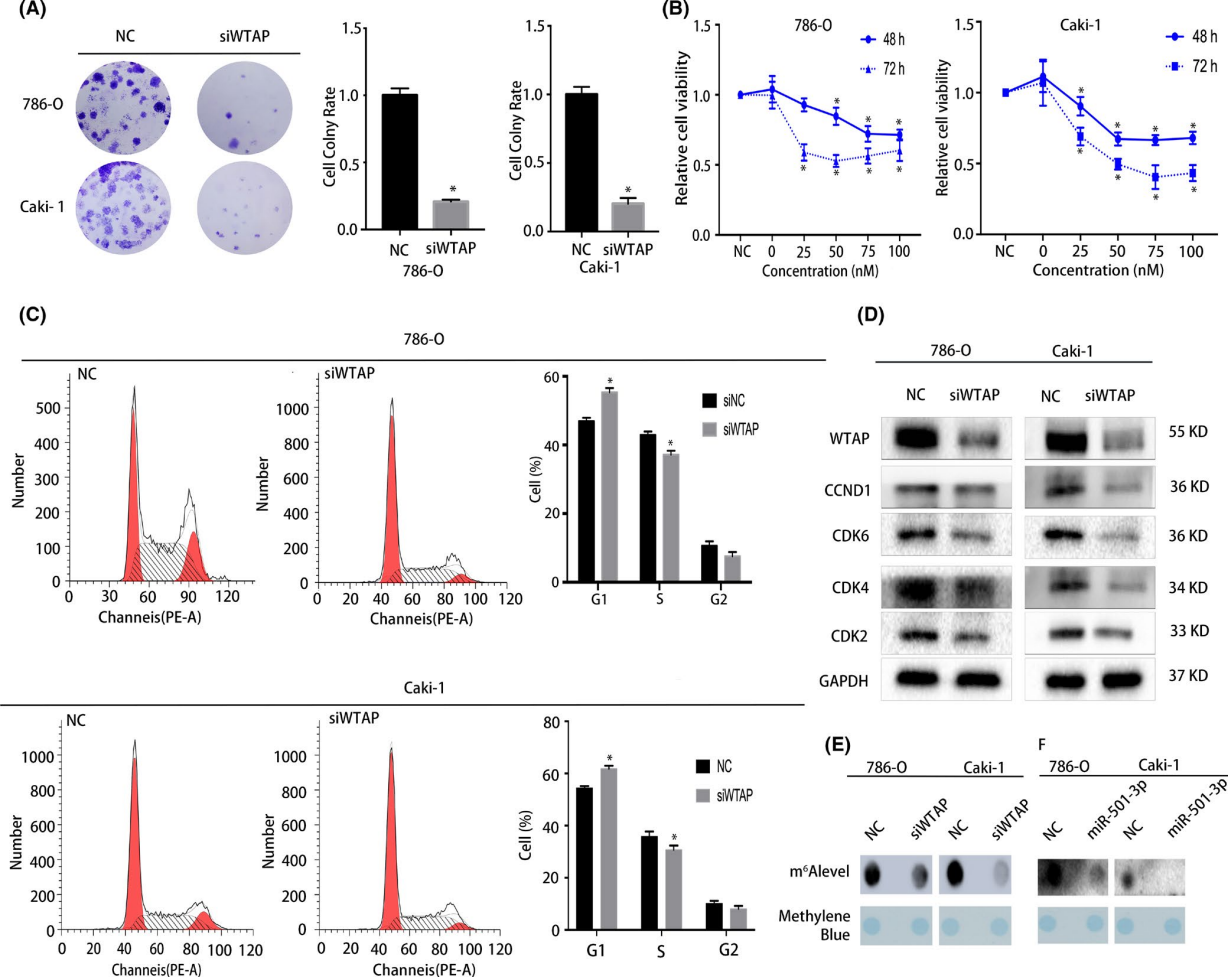
Silencing of the *WTAP* gene using siRNAs significantly inhibited the proliferation of renal cell carcinoma (RCC) cells. (A) Colony formation assay indicated knocking down *WTAP* reduces the colony‐forming ability of RCC cells. (B) CCK8 assay showed that as the concentration of the siRNA library in the transfected cell lines increased, the cell viability decreased. (C) Cell cycle analysis demonstrated that the proportion of cells in the G1 phase increased after knocking down *WTAP*. (D) Western blots confirmed that treatment with siWTAP inhibited the proliferation of RCC cells. (E) Dot blot assay demonstrated that the level of m^6^A decreased with the tapping of *WTAP*. (F) Dot blot assay demonstrated that the m^6^A level changed with the expression of miR‐501‐3p; Error bars represent the standard deviation (SD) obtained from three independent experiments. *p < 0.05

Given that *WTAP* is upregulated in RCC, we transfected RCC cells with siRNAs against the gene to analyze its effect. The CCK8 and colony formation assay results revealed that the knockdown of *WTAP* significantly inhibited the proliferation of the two cancer cell lines (Figure 5A,B). Furthermore, the flow cytometric results revealed that *WTAP* knockdown caused substantial G1/S arrest (Figure 5C). Subsequently, the western blot results also revealed the corresponding changes in the G1/S transition regulators, including CDK2, CDK4, CDK6, and CCND1 (Figure 5D).

### 3.6 miR‐501‐3p overexpression or *WTAP* knockdown changes the total RNA m^6^A level

As an m^6^A methyltransferase, WTAP has an important regulatory effect on mRNA methylation. We hypothesized that miR‐501‐3p targets *WTAP* directly to change the m^6^A levels, thereby regulating the progression of RCC. To verify our hypothesis, dot‐blot assays were used to detect changes in the m^6^A levels in the various cell lines transfected with either the miR‐501‐3p mimic or siWTAP. The results indicated that the total RNA m^6^A levels were markedly diminished in the miR‐501‐3p‐overexpressing or WTAP‐silenced cells (Figure 5E,F).

### 3.7 *WTAP* overexpression rescues the miR‐501‐3p‐induced suppression of kidney cancer cell proliferation

**FIGURE 6:**
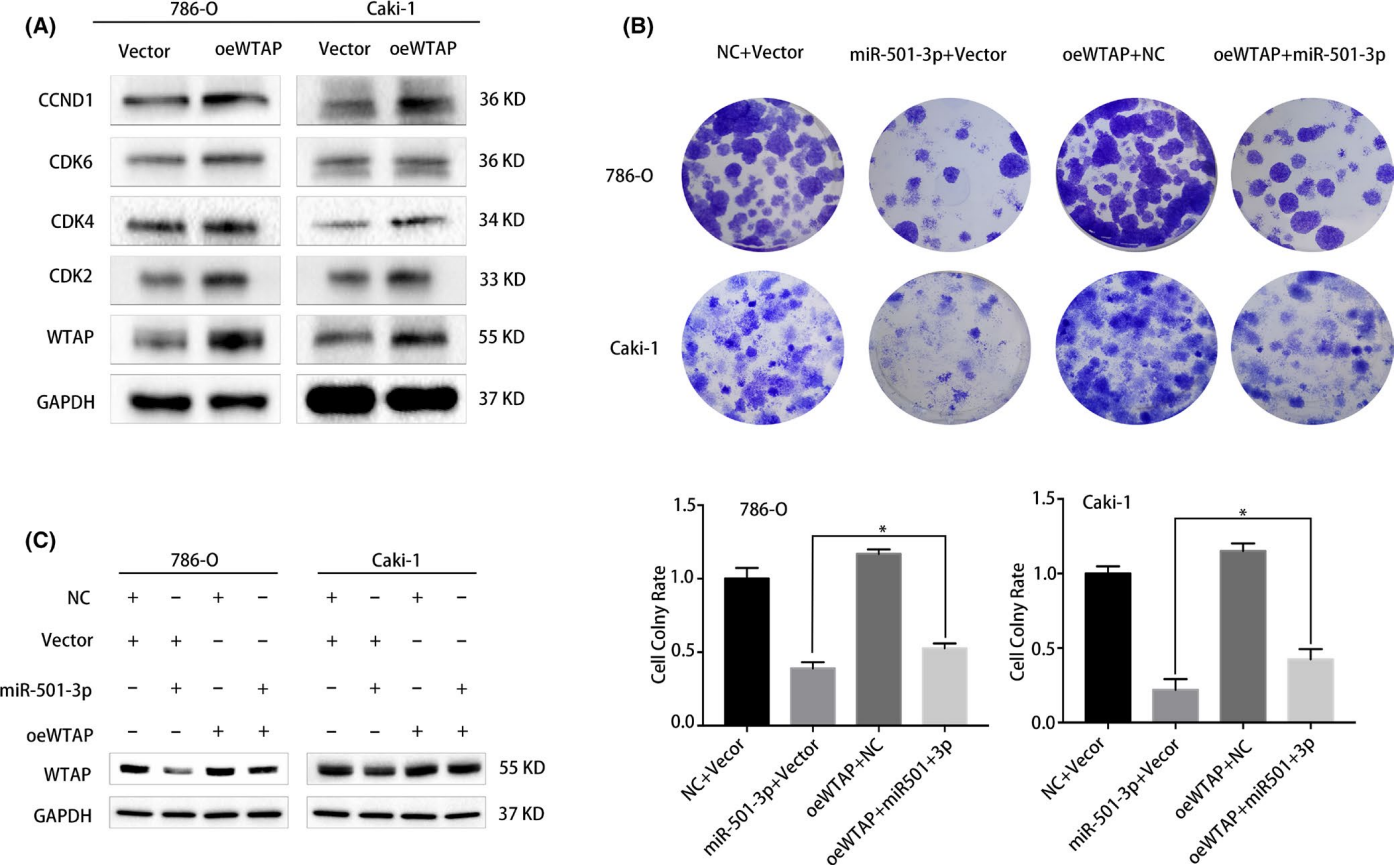
*WTAP* overexpression rescues the miR‐501‐3p overexpression‐induced suppression of renal cell carcinoma (RCC) cell proliferation. (A) Western blots analysis of the expression of corresponding G1/S transition regulators in RCC cell lines with WTAP overexpression. (B) Colony formation assay indicated that the co‐transfection of miR‐501‐3p mimic and *WTAP* plasmid reversed the proliferation inhibition induced by miR‐501‐3p overexpression in RCC cell lines. (C) Western blots showed that the co‐transfection of the miR‐501‐3p mimic and WTAP plasmid attenuated the WTAP plasmid‐induced suppression of miR‐501‐3p at the protein level in RCC cell lines. Error bars represent the standard deviation (SD) obtained from three independent experiments. *p < 0.05

The changes in *WTAP* expression suggested that its overexpression promoted the proliferative abilities of the two RCC cell lines (Figure 6A). To further evaluate the correlation between miR‐501‐3p and *WTAP*, plasmids carrying the miR‐501‐3p mimic and *WTAP* gene were used to conduct rescue experiments. According to the colony formation assay results, the proliferative abilities of the RCC cells were enhanced by *WTAP* overexpression, and the inhibition of cell proliferation induced by miR‐501‐3p overexpression was notably reversed by co‐transfection with the WTAP‐carrying plasmid (Figure 6B). As expected, these changes in the two cell lines were consistent with similar *WTAP* overexpression at the protein level (Figure 6C).

## 4 DISCUSSION

An increasing number of patients are being diagnosed with RCC owing to their adoption of an unhealthy lifestyle. Because the disease in many patients usually progresses beyond surgical treatment options when first diagnosed, the prognosis for RCC is generally poor.^2^, ^4^, ^5^ Because little is known about the progression and molecular mechanisms of RCC, the elucidation of these unknowns would be of great clinical significance for the primary diagnosis and treatment. MiRNAs are involved in various biological processes.^10^, ^11^, ^12^, ^13^, ^18^, ^19^, ^20^ Human miR‐501‐3p, which is located at chromosome position Xp11, has been found in different types of cancers, where it directly targets several genes, such as *FOS*, *MDFI*, *MyoD*, *LIN7A*, and α‐amino‐3‐hydroxy‐5‐methyl‐4‐isoxazolepropionic acid receptors (*AMPA*).^16^, ^17^, ^21^ MiR‐501‐3p overexpression can inhibit the proliferative ability of prostate cancer cells and induce G1 phase arrest.^22^ In addition, miR‐501‐3p promotes osteosarcoma cell proliferation by targeting *BCL7A* ^23^ and the miRNA can directly target the *RAS* oncogene *RAP1A* to repress non‐small cell lung cancer cell proliferation.^15^ In addition, downregulation of miR‐501‐3p has been reported in several types of human cancers.^15^, ^16^, ^22^ Although there are few formal studies on miR‐501‐3p in RCC, it can be expected that miR‐501‐3p exerts its function in RCC. According to the TCGA database and our clinical samples, miR‐501‐3p was markedly downregulated in RCC tissues than in normal renal tissues. In our study, miR‐501‐3p was shown to play an inhibitory role in the proliferation of RCC, and its expression in cancerous cells and tissues was notably downregulated relative to that in the normal cell and tissue counterparts. Naturally, miR‐501‐3p upregulation significantly repressed the proliferation of kidney cancer cells. Previous studies have shown that miR182‐5p contributes to renal cell carcinoma proliferation by activating the AKT/FOXO3a signaling pathway^10^ and that miR‐452‐5p promotes the proliferation of RCC by modulating SMAD4/SMAD7 signaling.^11^ The role of miR‐501‐3p in the progression of renal cancer is similar to that of the abovementioned miRNAs.^11^, ^12^, ^13^

We confirmed that *WTAP* is the target gene of miR‐501‐3p. The rescue experiment verified that miRNA overexpression could effectively repress WTAP‐induced cell proliferation. *WTAP* is a conserved nuclear protein that is a companion to the Wilms’ tumor gene (WT1),^24^ which is often mutated in Wilms tumors and is considered a tumor suppressor.^25^ The silencing of the *WTAP* gene is lethal to embryos.^26^ Moreover, *WTAP* facilitates the progression of hepatocellular carcinoma via m^6^A‐HuR‐dependent epigenetic silencing of *ETS1* ^27^ and promotes osteosarcoma tumorigenesis by repressing *HMBOX1* expression.^28^ These findings indicate that *WTAP* is related to several cellular processes, such as the cell cycle, selective splicing, X‐chromosome inactivation, apoptosis, and m^6^A modification.^27^, ^29^, ^30^, ^31^, ^32^ It is also considered as an oncogene that is positively correlated with the progression of malignant tumors.^27^, ^32^ *WTAP* is highly expressed in cells of hepatocellular carcinoma, osteosarcoma tumorigenesis, gastric cancer, and cholangiocarcinoma, where it is correlated with poor survival outcomes.^27^, ^28^, ^33^, ^34^ Previous findings have revealed that the expression of *CDK2* is closely related to that of *WTAP* in RCC. *WTAP* can promote *CDK2* mRNA stability by targeting the 3‐UTR of mRNA.^26^, ^35^ This was consistent with our experimental results. Therefore, we propose that miR‐501‐3p enhances the progression of RCC via the miR‐501‐3p/WTAP/CDK2 axis. In hepatocellular carcinoma, the miR‐139‐5p loss‐mediated *WTAP* activation contributes to its progression.^36^ Long noncoding RNA *DUXAP8* promotes pancreatic carcinoma progression via the miR‐448/WTAP/Fak signaling axis^37^ and miR‐155 accelerates the growth of human liver cancer cells by activating *CDK2* via targeting *H3F3A*.^38^ In addition, miR‐200c targets *CDK2* and suppresses tumorigenesis in RCC.^39^ These results suggest that miRNA, *WTAP*, and *CDK2* are jointly involved in the progression of various tumors, which is consistent with our experimental results showing that miR‐501‐3p enhances the progression of RCC via the miR‐501‐3p/WTAP/CDK2 axis. Additionally, *WTAP* has been shown to accelerate the degradation of target mRNAs by promoting the formation of m^6^A.^40^ As the most copious and reversible RNA modification process in eukaryotic cells, methylation involves ‘writer’ complexes, enzymes called ‘erasers’ that remove modifications, and ‘readers’ of the histone code.^41^, ^42^, ^43^, ^44^ Generally, m^6^A modification performs promotion or suppression functions in the tumorigenesis and progression of different cancers.

In this study, we explored whether *WTAP*, as a methyltransferase, plays a role in RCC by regulating the m^6^A levels. We showed that the silencing of *WTAP* reduced the total m^6^A level in cancer cells, and miR‐501‐3p overexpression produced the same results. However, in follow‐up experiments, we failed to substantiate the role of WTAP‐regulated m^6^A modification in RCC. Therefore, the specific mechanism through which miR‐501‐3p and *WTAP* interact to inhibit RCC requires further study.

In summary, we showed that miR‐501‐3p is a latent suppressor of RCC and is commonly downregulated in cancerous kidney cells and tissues, whereas its overexpression inhibits the progression of the disease. We further identified *WTAP* as the target gene of miR‐501‐3p in RCC. This study sheds light on the miR‐501‐3p‐mediated regulatory network in RCC and identifies molecular targets that could be useful for the early diagnosis and treatment of RCC.

## CONFLICT OF INTEREST

The authors declare no conflicts in this study.

## ETHICS APPROVAL STATEMENT

Twenty‐four paired renal cancer tissues and adjacent non‐tumor tissues were obtained from patients undergoing radical nephrectomy. The samples were collected between January 2013 and October 2013 at the First Affiliated Hospital, Zhejiang University School of Medicine (Hangzhou, P.R. China) after obtaining informed consent and the approval of the Clinical Research Ethics Committee of the First Affiliated Hospital, Zhejiang University School of Medicine (ID: IIT20200733A).

## Supporting information

Table S1

Table S2

## Data Availability

All data generated or analyzed during this study are included in this published article.
