# Supplementary material for: MicroRNA‐501‐3p inhibits the proliferation of kidney cancer cells by targeting WTAP
Source: Cancer Med. 2021 Sep 30;10(20):7222–32. doi: 10.1002/cam4.4157 (PMC8525086; doi:10.1002/cam4.4157)
Supplement: Supplementary file 1 — Table S1 [file CAM4-10-7222-s001.docx]

# Supplemental Table S1 Patients and tumor characteristics (n = 24)

| No. | Age | Sex | Pathologic Diagnosis | pT Stage | Fuhrman Grade |
| --- | --- | --- | --- | --- | --- |
| 1 | 55 | male | clear cell | T1b | Ⅱ |
| 2 | 55 | male | clear cell | T2a | Ⅱ |
| 3 | 36 | male | papillary | T1a | Ⅰ |
| 4 | 49 | female | clear cell | T2a | Ⅰ |
| 5 | 55 | male | clear cell | T3a | Ⅱ |
| 6 | 59 | female | clear cell | T1b | Ⅰ |
| 7 | 68 | female | clear cell | T3a | Ⅱ |
| 8 | 74 | male | clear cell | T1b | Ⅰ |
| 9 | 62 | male | clear cell | T1b | Ⅱ |
| 10 | 75 | male | clear cell | T1a | Ⅰ |
| 11 | 43 | male | clear cell | T1a | Ⅰ |
| 12 | 59 | male | clear cell | T3b | Ⅲ |
| 13 | 58 | female | clear cell | T1b | Ⅰ |
| 14 | 54 | female | clear cell | T3a | Ⅰ |
| 15 | 60 | male | clear cell | T4 | Ⅱ |
| 16 | 63 | female | clear cell | T1b | Ⅱ |
| 17 | 65 | female | clear cell | T1b | Ⅰ |
| 18 | 54 | male | clear cell | T3a | Ⅱ |
| 19 | 57 | male | clear cell | T1a | Ⅰ |
| 20 | 56 | male | clear cell | T2a | Ⅲ |
| 21 | 53 | female | clear cell | T1b | Ⅰ |
| 22 | 67 | male | clear cell | T2a | Ⅰ |
| 23 | 51 | female | clear cell | T1b | Ⅱ |
| 24 | 65 | male | clear cell | T1b | Ⅰ |
