# Supplementary material for: MicroRNA‐501‐3p inhibits the proliferation of kidney cancer cells by targeting WTAP
Source: Cancer Med. 2021 Sep 30;10(20):7222–32. doi: 10.1002/cam4.4157 (PMC8525086; doi:10.1002/cam4.4157)
Supplement: Supplementary file 2 — Table S2 [file CAM4-10-7222-s002.docx]

# Supplemental Table S2 The oligonucleotides used in this study.

| Name^a^ | Sequence (5’->3’) ^b^ |
| --- | --- |
| NC (sense) | ACUACUGAGUGACAGUAGA |
| siWTAP (sense) | GCGAAGUGUCGAAUGCUUATT  GGGUAUGCAGAGUACCAUUTT |
| hsa-miR-503-3p mimic (Sense) | AAUGCACCCGGGCAAGGAUUCU |
| NC（Sense） | UUACGUGGGCCCGUUCCUAAGA |
| SLMAP-F | TCAGGAGCGTCATGTCTACCT |
| SLMAP-R | CGTGGTTCCTTGATAGCACTTTG |
| hsa-miR-501-3p-F | AATGCACCCGGGCAAGGATTCT |
| CLIC4-F | TGAAAGCATAGGAAACTGCCC |
| CLIC4-R | GGTCAACAGTCGTCACACTAAA |
| MAPK14-F | CCCGAGCGTTACCAGAACC |
| MAPK14-R | TCGCATGAATGATGGACTGAAAT |
| WTAP-F | CTTCCCAAGAAGGTTCGATTGA |
| WTAP-R | TCAGACTCTCTTAGGCCAGTTAC |
| METTL2A-F | GCAGTCCTCGCCGATAAGAG |
| METTL2A-R | CTTCCGACCACTCCACATTGT |
| YTHDF2-F | AGCCCCACTTCCTACCAGATG |
| YTHDF2-F | AGCCCCACTTCCTACCAGATG |
| SAMD12-F | TGCCCATGCTGAAGGTATTAAAC |
| SAMD12-R | CGTAGCTGACTTAGCCGTCT |
| MLLT6-F | CTGCGTATGTTCGGACGAGAG |
| MLLT6-R | CACCTGAACGATGCCATAGCA |
| HSPA13-F | CCAGAGAGATGACGATCTTAGGA |
| HSPA13-R | TCACTTTAGGAGTAGGCAATGGT |
| GAPDH-F | GGAGCGAGATCCCTCCAAAAT |
| GAPDH-R | GGCTGTTGTCATACTTCTCATGG |
| U6 | TGCGGGTGCTCGCTTCGGCAGC |
| CDK2-F | CCAGGAGTTACTTCTATGCCTGA |
| CDK2-R | TTCATCCAGGGGAGGTACAAC |
| METP1-F | CATCCAGGGCTCGTACTTCTG |
| METP1-R | TCTCGCTTCGCCTTTTCATCT |
| U6-F | TGCGGGTGCTCGCTTCGGCAGC |
| pmirGLO primer | ACACGGTAAAACCATGAC |
| WTAP-WT-F | CGACTGTTTAAGAAATTTGTGTGCATAGTTTCAGTTTTTATGAACTG |
| WTAP-WT-R | TCGACAGTTCATAAAAACTGAGTAACTATGCACACAAATTTCTTAAACAGTCGAGCT |
| WTAP-MUT-F | CGACTGTTTAAGAAATTTGTCACGTATCTTACTCAGTTTTTATGAACTG |
| WTAP-MUT-R | TCGACAGTTCATAAAAACTGAGTAAGATACGTGACAAATTTCTTAAACAGTCGAGCT |

^a^ F, forward primer; R, reverse primer.

^b^ Restriction sites are in bold
